# Recommendations for Haemodynamic and Neurological Monitoring in Repair of Acute Type A Aortic Dissection

**DOI:** 10.1155/2011/949034

**Published:** 2011-07-14

**Authors:** Deborah K. Harrington, Aaron M. Ranasinghe, Anwar Shah, Tessa Oelofse, Robert S. Bonser

**Affiliations:** ^1^Department of Cardiac Surgery, UHB NHS FT, Edgbaston, Birmingham B15 2TH, UK; ^2^School of Clinical and Experimental Medicine, University of Birmingham, Edgbaston, Birmingham B15 2TT, UK; ^3^Department of Cardiac Anaesthesia, UHB NHS FT, Edgbaston, Birmingham B15 2TH, UK

## Abstract

During treatment of acute type A aortic dissection there is potential for both pre- and intra-operative malperfusion. There are a number of monitoring strategies that may allow for earlier detection of potentially catastrophic malperfusion (particularly cerebral malperfusion) phenomena available for the anaesthetist and surgeon. This review article sets out to discuss the benefits of the current standard monitoring techniques available as well as desirable/experimental techniques which may serve as adjuncts in the monitoring of these complex patients.

## 1. Introduction

This review encompasses our opinions and recommendations regarding haemodynamic and neurological monitoring during the conduct of acute type A dissection repair (ATAAD) and centre around the detection and avoidance of intraoperative malperfusion. The review covers the conduct of operation and monitoring that is relevant for most institutions that are not major tertiary aortic centres. We have not considered pharmacological and anaesthetic techniques of management of the dissection patient as this topic is beyond the remit of this review.

**Figure 1:**
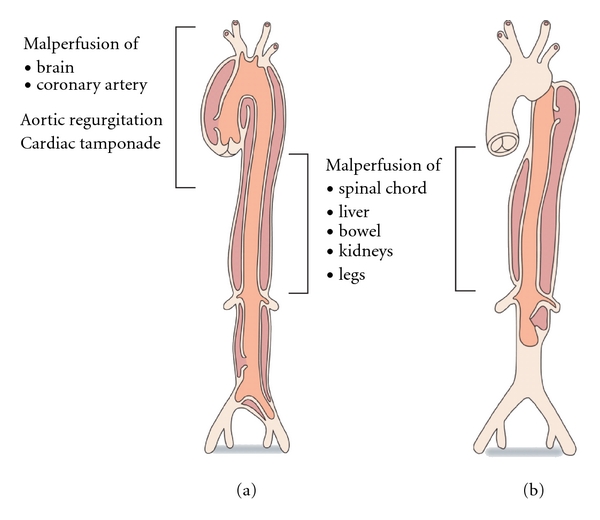
Subtypes and complications of aortic dissection, (a) type A and (b) type B aortic dissections. Type A encompasses Debakey I (ascending aorta only and therefore less potential for malperfusion phenomena) and II and type B Debakey III classifications. Reproduced with permission from Golledge and Eagle [4].

The pragmatic Stanford classification of aortic dissection assigns the descriptor type A to those dissections involving the ascending aorta (which in the majority of cases require emergency surgical repair) while type B categorises those dissections not involving the ascending aorta (which are often initially managed medically). ATAAD incorporates types I and II of the DeBakey classification (type I ascending and distal aorta and type II ascending aorta only) while Stanford type B dissections, which arise usually distal to the origin of the left subclavian artery are analogous to DeBakey-type III lesions (Figure 1). Within the type A category, the DeBakey-type I dissection can be regarded as more sinister than type II because of the increased propensity for malperfusion phenomena and patency of a residual false lumen after any surgical repair. ATAAD is highly lethal when managed conservatively. Historically, mortality has been approximated at 1-2% per hour following symptom onset with up to 90% mortality within 30 days [1–3]. This excessive mortality is due to intrapericardial rupture with tamponade, acute severe aortic regurgitation with heart failure secondary to acute left ventricular volume overload, malperfusion of the coronary, central nervous or visceral circulation, and rupture beyond the pericardium.

 Despite excellent results from individual surgeons and expert centres [6, 7] and advances in anaesthetic, perfusion, and surgical techniques [8], in general, operative outcomes for ATAAD have remained static with mortality rates from large databases that perhaps better reflect “real-world” practice still around 15–30% [2, 9–13]. Despite this, emergency operative repair converts a 90% mortality into a 70% survival chance and as such currently remains the gold standard of care. 

## 2. Aims of Surgery

The aim of surgical intervention for ATAAD is to manage the potentially fatal complications associated with it. These are

- intrapericardial rupture and tamponade,
- malperfusion phenomena (coronary, neurological, and visceral),
- acute aortic valve regurgitation.

These aims are achieved by replacement of the ascending aorta (including the aortic root if required) with resuspension/replacement of the aortic valve and excision of the proximal entry tear if possible and obliteration of the distal false lumen. 

## 3. The Propensity for Malperfusion: The Crux of Haemodynamic Monitoring

**Figure 2:**
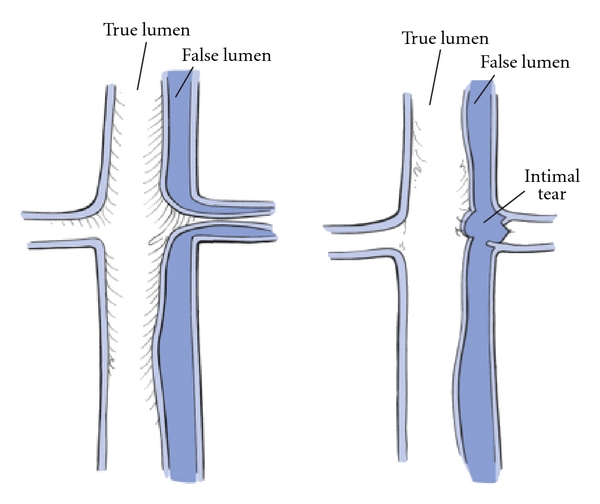
Malperfusion phenomena associated with acute type A dissection, (a) malperfusion is secondary to true lumen compression by the false lumen and (b) occlusion of the branch vessel by extension of the false lumen. Reproduced with permission from Reece et al. [5].

Malperfusion may be defined as a compromise of flow within the aorta or its branch vessels which may lead to end-organ ischaemia (Figure 2). The predominant aetiology relates to pressurisation of the false lumen without a reentry tear. The pressurised false lumen then partially or completely obstructs true luminal flow, jeopardising end-organ perfusion. Usually, this is a dynamic phenomenon. This means that during ventricular systole, preferential false luminal perfusion via a large entry tear occurs and the true lumen collapses leading to malperfusion. Although, pressurisation abates during diastole, end-organ perfusion is still compromised. Other types of malperfusion phenomena may occur with a fixed or static obstruction occurring when the larger false lumen is thrombosed. In other circumstances, thrombosis in low-flow dissected arteries may itself lead to thrombosis while in others complete transection of the intima may lead to intussusception into a vessel with compromise of flow. It is important to differentiate between radiological and clinical malperfusion. Radiological malperfusion is more common and represents apparent embarrassment of flow to branch vessels. However, the prognostic relevance of malperfusion is determined by evidence of end-organ ischaemia and its clinical effects. Therefore, while some embarrassment of innominate artery flow may be detected on computerised tomography, it is only when this is accompanied by clinically apparent neurological sequelae that patient outcome is affected.

The DeBakey classification adds additional information of relevance to malperfusion in ATAAD. A DeBakey-type II dissection, restricted to the ascending aorta may cause coronary, cerebral and right upper limb malperfusion but by definition, in the absence of aortic luminal compromise, should not contribute to more distal malperfusion phenomena. In contrast, a DeBakey-type I dissection that extends beyond the aortic arch has the additional potential to generate more distal malperfusion phenomena including the left upper limb, spinal cord, viscera and kidneys, and lower limbs. 

## 4. When Does Malperfusion Occur?

### 4.1. Presurgery Malperfusion

Malperfusion phenomena may already exist prior to surgery and the timing of their management before, during or after aortic repair is a matter of great debate [14–16]. Moreover, the known presence of malperfusion will affect monitoring strategy. In the International Registry of Aortic Dissection (IRAD) database patients presenting with clinically detectable pulse deficits (carotid or peripheral) have been demonstrated to have a worse prognosis. Pulse deficit has been shown to be an independent predictor of in-hospital mortality, and mortality rate increases with the number of pulse deficits detected [17].

Malperfusion phenomena may worsen during anaesthesia, and it is thus important that anaesthesia should be expeditious, and essential monitoring that could detect any deterioration, particularly of cerebral perfusion status, is rapidly instituted. During surgery there are several crucial timepoints where *de novo* malperfusion may occur and rapid detection is needed to alter surgical strategy and prevent fatality. 

### 4.2. Malperfusion at Institution of Cardiopulmonary Bypass

Arterial cannulation at any peripheral sites for example, femoral artery or axillary artery, can, on institution of cardiopulmonary bypass (CPB), precipitate or worsen malperfusion. The retrograde arterial flow towards the heart, even when the cannula lies within the vessel's true lumen, may lead to differential false luminal dominance via a primary or secondary tear, false luminal pressurization, and cerebral, cardiac, or other organ malperfusion. Cerebral malperfusion, at normothermia and during cooling, if sustained will lead to neurological injury. Thus, an otherwise flawless operative strategy with an adequately performed distal anastomosis performed under conditions of hypothermic circulatory arrest (HCA) with or without perfusion adjuncts will have an unsatisfactory outcome unless the phenomenon is detected and corrected. Historically, the femoral artery has often been used for arterial cannulation due to ease of access, size, and ability to achieve adequate flow rates [18, 19]. However, retrograde femoral perfusion carries the risk of pressurisation of the dissection false lumen, leading to compromise of true luminal flow and malperfusion [20–22]. Increasingly the right axillary or subclavian artery has been used for arterial cannulation with well-documented results [23]. Commonly, an 8 mm graft is anastomosed to the artery in an end-to-side fashion which then allows antegrade blood flow during CPB without further aortic manipulation. It may also reduce emboli from an atheromatous aorta or retrograde femoral arterial flow. Such cannulation may have a lower propensity to induce cerebral malperfusion at this stage of the procedure [24] but even this perceived lower risk of malperfusion and retrograde embolism may result in further dissection propagation and malperfusion [25, 26]. Direct aortic cannulation, carotid artery cannulation, and transapical left ventricular-aortic cannulation have all been utilised to try and ensure true luminal flow [27–32]. No method is exempt from generating malperfusion at commencement of CPB. Thus, the institution of CPB is a timepoint for extravigilant monitoring. Detection of malperfusion at this point should prompt the surgeon to change the arterial cannulation site immediately to ensure true luminal flow. 

### 4.3. Malperfusion Occurring after Aortic Cross-Clamping

Many surgeons clamp the ascending aorta after the institution of CPB in order to proceed with the proximal aortic repair. Others prefer not to clamp and to undertake a distal anastomosis first without instrumenting the aorta [34]. Clamping may be undertaken even when there is an intention to proceed to hypothermic circulatory arrest (HCA) to undertake an open distal anastomosis. Although comparative studies have shown little difference in patient outcome, placement of an ascending aortic cross-clamp may obstruct communication between true and false lumina leading to false luminal pressurisation and as a consequence, cerebral malperfusion or even aortic rupture. Thus, if clamp placement during cooling is the preferred surgical technique, vigilant monitoring at this time is essential and the proximal aorta should not be opened until surgeon and anaesthetist are satisfied that brain perfusion at least is not compromised. 

### 4.4. Malperfusion Postdistal Aortic Anastomotic Construction

A further hazardous timepoint occurs at reinstitution of CPB flow following construction of the distal aortic anastomosis. When the distal anastomosis is constructed, the surgeon attempts to reapproximate the dissected aortic wall and close entry to the false lumen. If peripheral arterial cannulation has been used, there is again the potential for perfusion of the false lumen now closed at the site of the anastomosis. Thus depending upon the position of the anastomosis, false luminal pressurisation and vessel compromise may occur. For this reason, it is recommended that orthograde distal reperfusion is instituted either directly via the prosthetic graft or via a graft side-arm.

For these reasons it is important to have several intraoperative monitoring techniques in order to continuously and rigorously evaluate cerebral perfusion and allow changes to operative decision making should a problem occur. 

## 5. The Impact of Cerebral Malperfusion

**Figure 3:**
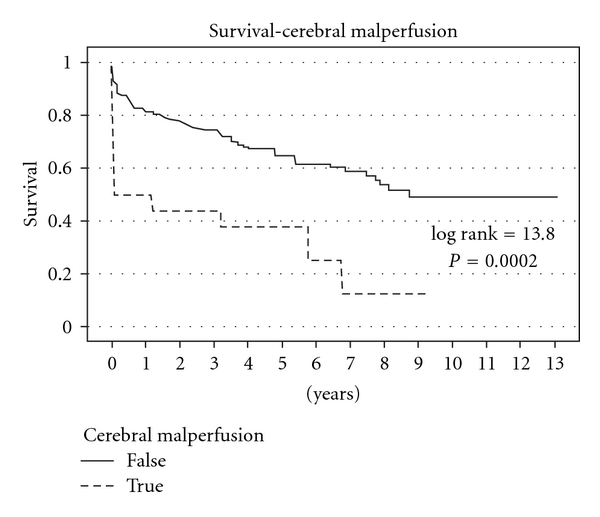
Kaplan Meier survival curves of actuarial survival of patients with cerebral malperfusion syndrome undergoing acute type A aortic dissection repair. Reproduced with permission from Geirsson et al. [36].

The incidence of neurological compromise evidenced by cerebral ischaemia and malperfusion at presentation is quoted to be between 5–14% [9, 35–37] and is associated with poor early and midterm outcomes (Figure 3) [35, 36, 38]. Neurological injury may be secondary to hypotension, malperfusion, or thromboembolic phenomenon. In approximately half of patients with neurological deficits, these are temporary, and in a third of patients the presenting symptom may not be associated with chest pain, which further complicates the diagnosis [39]. Not only are patients with ATAAD subject to neurological compromise at presentation, but surgery itself for treatment of ATAAD is associated with permanent neurological injury rates of as high as 26% depending on the age of the patient with temporary deficit rates of up to 32% [40–42]. A shorter time between symptom onset and surgical reperfusion is associated with improved outcomes particularly if presentation is less than five hours and has a reasonable prospect of limiting stroke progression [43, 44]. A precise cutoff time above which recovery is unlikely is as yet undefined. A ten-hour threshold, below which neurological conservation and recovery may be higher than has been suggested [37, 45] but reperfusion is no guarantee that stroke will be prevented or that full independent rehabilitation will occur [35, 37, 46]. Conscious patients who have either a temporary or permanent neurological deficit have similar outcomes and long-term survival to patients presenting without a neurological deficit and in more than half, the deficit can be expected to improve [36, 37]. However, for patients who are either moribund or comatose due to the consequences of established cerebral malperfusion, operative mortality may be excessively high and nonoperative intervention in such cases may be justifiable. 

## 6. Cerebral Protection

The generally accepted repair of ATAAD involves proximal aortic replacement with either aortic valve resuspension or replacement and an open distal anastomosis employing HCA as the mainstay of cerebral protection. The main premise of HCA is that by using profound hypothermia (20°C or below) it is possible to reduce cerebral metabolic requirements enough to allow a period of circulatory arrest during which it is technically feasible to construct the distal anastomoses [47]. HCA enables visualisation of the aortic arch to inspect for intimal tears. However, HCA only allows for limited periods of repair as the rate of adverse cerebral outcomes is closely related to time and the temperature at which arrest occurs [48–51] (Table 2). Adjunctive cerebral protective techniques have been developed in an attempt to reduce the risks associated with HCA and to prolong the potential “safe” duration of arrest. The first adjunctive technique to be used widely was retrograde cerebral perfusion (RCP) which entails retrograde perfusion via the superior vena cava (SVC) during the HCA period [52, 53]. The potential benefits of RCP are of brain cooling, cerebral perfusion, and elimination of embolic debris and metabolites. One of the complications, however, was a potential increase in the incidence of cerebral oedema. Thus it is important to monitor the pressure delivered via the SVC usually measured via the central venous line with a target of 25 mm Hg [54]. In many centres, RCP has been abandoned due to lack of evidence of benefit [25, 55]; however, some centres have documented improved outcomes versus HCA alone [56–58], and it remains a centre-specific adjunct used in an attempt to extend the safe duration of cerebral circulatory arrest.

The more common adjunctive technique now used when surgery is performed on the aortic arch is selective antegrade cerebral perfusion (SACP), which entails selective cannulation of the head and neck vessels, providing either unilateral cerebral perfusion or bilateral perfusion if both innominate and left common carotid are cannulated, either with or without snaring of the left subclavian artery. SACP is intuitively more physiological than RCP, and may provide both cooling and perfusion to the brain, although there were initial concerns over a potential for increase in embolic phenomena. The head and neck arteries are then able to be perfused for the majority of the corporeal arrest period apart from the time taken to insert and remove the cannulae and place the final few sutures. SACP has been demonstrated to reduce the cerebral metabolic deficit accrued following a period of HCA [59] and is widely associated with improving clinical outcomes [60–63]. As yet optimal SACP perfusate characteristics such as temperature, pressure, and flow rates remain undetermined and under investigation [64]. There is significant debate regarding the use of unilateral or bilateral SACP. Unilateral cannulation of either the innominate or the left common carotid only is technically simpler and involves less time and cumbersome equipment in the operative field. It has been demonstrated to produce satisfactory results [65] but has mainly been reported in the context of short arrest durations. Also, it relies upon a complex pre- and perioperative neurological monitoring strategy in order to detect and manage malperfusion, the scope of which is beyond most centres dealing with emergency ATAAD. Unilateral SACP has the potential to result in malperfusion if the patient has an anatomical variation of the Circle of Willis. In cadaveric studies, between 40 and 50% of the population have anatomical variations, which may be clinically significant in 14 to 40% of cases [66]. There have been no randomised studies between unilateral and bilateral cerebral perfusion, though most authors would agree that for emergency procedures and those with an expected long duration of HCA and SACP, bilateral perfusion is probably a safer option [67].

Over the past decade although an increasing number of cerebral protective techniques have become available, each with the potential to reduce the risk of neurological injury [41]. As yet there have been no universally accepted standards of both haemodynamic and neurological monitoring for repair of ATAAD. The aim of this review is to summarise the available evidence with regard to currently utilised monitoring techniques in ATAAD and to discuss potential novel techniques. 

## 7. Aims of Monitoring in ATAAD

Aside from providing vital information with regard to haemodynamic status and conduct of the operation, one of the primary reasons for continuous and detailed monitoring in patients undergoing surgery for ATAAD is to alert anaesthetic, surgical, and perfusion staff to ongoing events and potential catastrophes that may occur during surgery. This enables the teams involved to be aware of potential complications, formulate plans for their management, and be alerted once the patient leaves the operating theatre to problems that may be encountered on the intensive care unit.

There are a number of monitoring techniques employed during the operative management of ATAAD. Due to the emergency nature of this condition, there is very little randomised data providing evidence for such monitoring therefore the following review combines both published available evidence along with our experience-based recommendations in developing practicable monitoring techniques for this condition. From our experience we believe some are essential and others desirable; these are listed in Table 1.

All patients requiring emergent surgery for ATAAD repair should have a minimum standard of monitoring including electrocardiography, arterial oxygen saturations, capnography, arterial blood pressure, urine volumes, central venous pressure, as well as peripheral and core temperatures. 

## 8. Essential Monitoring

### 8.1. Electrocardiography

Monitoring of the electrocardiogram (ECG) gives pertinent information to the teams involved in care of the patient with ATAAD. As well as giving basic information on heart rate and rhythm, ECG monitoring with ST segment analysis may allow for diagnosis of coronary malperfusion in the perioperative phase and after the operation is completed give important information with regard to the adequacy of coronary perfusion if root replacement with coronary reimplantation is required. 

### 8.2. Temperature Monitoring

Temperature regulation with profound cooling is an essential component of surgery for ATAAD with open distal anastomoses being performed under hypothermic circulatory arrest (HCA). There are a number of temperature sites that can be measured as surrogates of brain temperature. If inadequate brain cooling is performed then the “safe” duration of HCA may not be as long as perceived. There are a number of potential sites at which temperatures may be obtained during cardiac surgery. These include arterial inflow, nasopharyngeal, oesophageal, bladder, skin, rectum, and tympanic membrane. Whilst surrogates of brain temperature, some of these sites may not accurately reflect true brain temperature. Perhaps the best surrogate of brain temperature (and the most invasive) is jugular bulb temperature [68]. In a study by Kaukuntla et al., nasopharyngeal temperature lagged behind arterial inflow and jugular bulb temperature during cooling for HCA with these temperatures all equilibrating at 15°C. During the rewarming phase both arterial inflow and nasopharyngeal temperature lagged behind jugular bulb temperature and the authors concluded that nasopharyngeal temperature is able to act as a safe surrogate for brain temperature although peripheral temperature measurements underestimate brain temperature and care is therefore required to avoid hyperthermic arterial inflow [69]. Partial rewarming utilising prolonged hypothermia has been demonstrated experimentally to have a better outcome when compared to normothermia [70] and animal studies after HCA have confirmed a worse neurological outcome when rewarmed to 40°C compared to 34°C. Most authors recommend the discontinuation of rewarming at a temperature of 36 to 37°C [71, 72]. We would recommend rewarming to an NP temperature of 36°C with a maximum arterial inflow of 37°C. 

### 8.3. Choice of Arterial Line

Due to the potential for malperfusion at varying sites in the aorta, it is important to monitor pressure at more than one site. The brain is the organ most sensitive to malperfusion and therefore detection of this is crucial. Innominate artery blood flow is essential for cerebral perfusion and a simple indicator of this is right radial artery pressure. Depending on the position of the intimal tear, malperfusion of the innominate artery will result in a reduction in right radial pressure which should be closely monitored to detect any potential changes in cerebral perfusion. Malperfusion of the left subclavian artery will result in reduced left radial pressure.

Therefore, the use of bilateral arterial line monitoring one placed proximal and the other distal to the aortic arch is strongly recommended. Changes in the arterial pressures monitored may alert the operating team to malperfusion phenomenon occurring across the aortic arch. These may be a combination of bilateral radial, or right radial and femoral lines. There is some debate whether the arterial monitoring site distal to the arch should be left radial or a femoral artery but there is no data to say which is preferable. Left arm monitoring in the presence of preoperative left subclavian malperfusion will not provide reliable pressure monitoring. Conversely, a distal femoral line may frustrate cannulation site selection, and if iliac malperfusion is present may give false readings of intra-arterial pressure. For this reason our own practice is to use 3-site monitoring when possible; right radial, left radial, and left femoral. This is partly dictated by the surgical choice of arterial cannulation; in our practice the most common scenario is of right axillary or right femoral artery cannulation for arterial inflow to establish cardiopulmonary bypass with bilateral radial arterial lines for monitoring. Where femoral cannulation is used, if pressurisation of the false lumen occurs resulting in cerebral malperfusion, a reduction in right radial pressure may be observed and expeditious alternative arterial cannulation needs to be performed, the most common scenario here is to rapidly transect the ascending aorta, remove the femoral arterial cannula, and place it into the true lumen of the open ascending aorta. An alternative strategy is to use a transapical approach via the left ventricle [73, 74]. Right axillary artery cannulation may carry a theoretically lower incidence of malperfusion but may still propagate the dissection. When the right axillary artery is cannulated directly, right radial artery monitoring no longer provides an index of innominate artery and thus right carotid pressure. When cannulated via an end-to-side graft, radial artery pressures may be increased spuriously and intraoperative pressure monitoring then becomes dependent upon monitoring at other sites, that is, left radial or femoral pressure. For this reason, pressure monitoring alone, when right axillary cannulation is used, is insufficient to monitor for evidence of malperfusion. It is important to heed and interpret any arterial line which reveals a low pressure and seek a cause. 

### 8.4. Transoesophageal Echocardiography

The use of transoesophageal echocardiography (TOE) has become commonplace in cardiac surgery, and many would regard it as an essential monitoring technique during surgery for ATAAD as well as being able to guide diagnosis [75–77]. Caution should be exercised on passage of the TOE probe as there is potential for acute haemodynamic compromise on insertion. In the face of haemodynamic instability, insertion of the TOE probe should be deferred until the patient is haemodynamically stable. In the preoperative phase, TOE [73, 78] can be used to assess the presence of pericardial fluid, aortic valvular competence, and regional wall abnormalities resulting from coronary ischaemia [79, 80] as well as also being a diagnostic tool in detecting the aortic dissection flap. It can also be used to assess the presence of atheroma in the descending aorta which is important if femoral arterial cannulation is planned and the IRAD group has demonstrated that TOE is able to add prognostic information above that gained from clinical risk factors at diagnosis [81]. In addition, TOE has been used to monitor cerebral and visceral malperfusion by direct visualisation of arch and visceral vessels to alert surgeon and anaesthetist to the development of malperfusion phenomena [82, 83]. Furthermore, Voci et al. have reported a technique of albumin microbubble injection via a side-port in the bypass circuit to aid in detection of true/false lumen detection at the time of institution of retrograde femoral perfusion. Detection of isolated false lumen perfusion utilising TOE can facilitate alternative cannulation strategies to minimise potential complications [84]. In the perioperative phase it may be used to position a femoral venous drainage cannula. after repair and separation from CPB, it is used to assess the competence of aortic valve resuspension, assess cardiac function, regional wall motion abnormality, and filling status. 

## 9. Intraoperative Blood Gas and Lactate Measurement

All patients undergoing surgery for ATAAD will undergo routine sampling of arterial blood gases via the in-dwelling arterial line. Information available from this includes ventilatory status, haemoglobin, and haematocrit estimation as well as acid-base status. Mixed venous oxygen saturation sampled from the cardiopulmonary bypass circuit gives an indication of global organ perfusion. Evidence of ongoing profound lactic acidosis may alert the team to the possibility of malperfusion phenomenon either related to limb or visceral ischaemia [85]. Examination of the peripheral circulation may demonstrate peripheral ischaemia, potentially secondary to femoral artery cannulation. In extreme circumstances, extension of the median sternotomy via laparotomy may be required to assess and address visceral ischaemia. 

## 10. Desirable Monitoring

### 10.1. Pulmonary Artery Catheter

Pulmonary artery catheterisation for thermodilution cardiac output and mixed venous oxygen saturation monitoring is highly desirable, but the timing of catheter flotation is discretional and should not delay surgery in a haemodynamically compromised patient. However, during line placement, expeditious insertion of an additional sheath for later pulmonary artery catheterisation is our preferred technique. Recent evidence from a meta-analysis in patients undergoing intermediate and high risk surgery [86] shows that preemptive haemodynamic optimization with a PAC may reduce mortality in some high-risk settings, but we would stress that preoperative optimization with a PAC is not always appropriate in the case of acute type A aortic dissection, where the priority is emergency surgery which should not be delayed. Flotation of a PAC is, however, desirable after separation from cardiopulmonary bypass when it can be used to best direct volume replacement and inotrope/vasoconstrictor requirements. Time spent undertaking preemptive haemodynamic optimisation is inappropriate if the cause of instability is tamponade or coronary malperfusion. In those situations we believe that rapid surgical relief of tamponade and coronary reperfusion are most likely to achieve better patient outcomes. 

### 10.2. Continuous Intra-Arterial Blood Gas Analysis

Technology is available for continuous intra-arterial blood gas analysis [87]. During cardiac and aortic surgery this technology has been demonstrated to be accurate and allow for early detection of changes in acid-base status [88, 89]. Although these technologies are not commonplace, they may allow for more detailed monitoring and recognition of malperfusion in patients undergoing surgery for ATAAD.

The vast majority of desirable monitoring techniques fall under the category of neurological monitoring. These techniques are mainly utilised and unlikely to be found outside large specialised aortic centres with an interest in neurological protection during aortic surgery.

In order to optimise cerebral protection during surgery for repair of ATAAD, whether or not standard HCA or adjunctive SACP are being used, it is important to monitor surrogates of cerebral blood flow and metabolism. There are now a number of techniques available for such purposes. These techniques require varying degrees of expertise. 

## 11. Surrogate Measures of Cerebral Flow and Metabolism

### 11.1. Transcranial Doppler

Transcranial doppler (TCD) is used to measure middle cerebral artery velocity (MCAV) via an acoustic window in the temporal bone. MCAV is used as a surrogate for cerebral blood flow and may be useful in arch surgery using RCP or SACP to monitor changes in blood flow. Changes in TCD velocity mirror changes in CBF but do not provide a direct measurement of CBF [90]. It is a noninvasive technique, using Doppler ultrasound, with the ability to obtain reasonable signals in 95% of individuals. It does require a limited amount of training, however, and for this reason its use is largely limited to research studies [59, 91]. On occasion, reversal of MCAV signalling may be detected in RCP [92] and in this circumstance has been used as an index of RCP adequacy. In a nonrandomised study, TCD utilised as an adjunct in patients undergoing ATAAD repair altered operative cannulation strategy and RCP perfusion in 28.5% and 78.6% of cases, respectively. Patients managed with TCD were also noted to have a reduced incidence of temporary neurological deficit [91]. TCD is not routinely used in ATAAD surgery for several reasons: (i) it requires a level of expertise and experience for correct signal acquisition, (ii) placement may be time consuming and inappropriate in ATAAD, (iii) loss of signal may occur due to minor displacement of probes and intraoperative correction once on bypass is difficult, and (iii) during periods of low flow, signals may be relatively poor and it may be difficult to differentiate between cerebral hypoperfusion and technical issues. However, as technology improves it can be expected that cerebral perfusion monitoring will play an increasing role. 

### 11.2. Orbital Ultrasound Monitoring

Doppler imaging allows for visualisation of orbital vessels [93]. It is noninvasive, inexpensive, and able to provide realtime information. Reductions in central retinal artery flow have been demonstrated to be associated with carotid artery stenosis [94], and maximal velocity within the CRA is correlated to perfusion pressure [93]. The absence of flow within the CRA and retrobulbar vessels has been demonstrated to be related to both transient and permanent neurological deficit. Absence of CRA flow for more than 100 minutes is associated with temporary neurological deficit and in a parallel with the data associated with neurological injury following 40 minutes of HCA, permanent neurological deficit has been observed in patients with an absence of retrobulbar flow for similar time periods [95]. Orbital ultrasound monitoring may have a role in monitoring during periods of selective cerebral perfusion with recommendations that perfusion pressures should be maintained to allow for detectable orbital Doppler flow. However, currently, this technique is not in widespread use. 

### 11.3. Electroencephalography

The electroencephalogram (EEG) represents electrical activity within the cerebral cortex. In noncardiac surgery it has been demonstrated to be both a sensitive and specific means of detecting cerebral hypoperfusion and ischaemia [96–98]. It is used in some aortic centres in order to monitor cerebral metabolic activity during aortic surgery [78, 99, 100], but may not be in common use during the emergent situation of ATAAD. During cooling, EEG activity is seen to decrease until electrocerebral silence occurs usually around temperatures of 15–18°C, or after 45–50 minutes of cooling. Alterations to EEG activity prior to this may indicate interruption to cerebral blood flow due to malperfusion allowing for early detection of malperfusion but once electrocerebral suppression has been achieved by cooling and anaesthesia, EEG monitoring cannot provide further guidance on the possibility of ongoing ischaemia; an important technical limitation. Technically, however, use of EEG monitoring is largely limited by its availability and logistical issues and is not always suitable for emergency cases. It is also affected by interference from diathermy and other electrical equipment. However, if routinely and expeditiously available, it should be used. 

### 11.4. Near-Infrared Spectroscopy

Near-infrared spectroscopy (NIRS) provides continuous monitoring of regional cerebral tissue oxygen saturation (rCSO_2_), it is noninvasive and a simple means of monitoring trends in rCSO_2_. In combination with multisite pressure monitoring it is our preferred technique for cerebrospecific monitoring in ATAAD. It involves placement of two single use adhesive patches on the forehead, avoiding the sinuses and temporal muscles. Near-infrared light is largely absorbed in tissues by oxygenated and deoxygenated haemoglobin. Absorption is proportional to the concentration of the haemoglobin and thus tissue saturation can be derived. The majority of the blood in the region of the cortex monitored is venous blood. Therefore, changes in rCSO_2_ reflect imbalances in either oxygen supply or consumption. Mean tissue oxygen saturation are estimated to be approximately 60–70%. It has demonstrated good reproducibility, and is being used more widely. In coronary artery bypass surgery use of monitoring of rCSO_2 _ avoids cerebral desaturation and is associated with a reduction of major organ dysfunction. In this trial by Murkin et al., the control group of patients had rCSO_2 _ monitoring but without intervention. In the intervention group most commonly fall in rCSO_2 _ was corrected by increased pump flow, increased mean arterial pressure, or normalization of CO_2_ changes in levels of CO_2_ may be pertinent in NIRS as the cerebral vasculature is sensitive to changes in levels of CO_2_ and changing concentrations can lead to alterations in cerebral blood flow which may be detected by NIRS. A significantly greater number of patients in the control group experienced a composite endpoint of major organ morbidity or mortality. Control patients also had longer periods of cerebral desaturation and longer intensive care unit stay as well as a trend towards increased overall increase in length of hospitalization. Patients with major organ morbidity or mortality had lower baseline and mean rCSO_2_ more episodes of cerebral desaturation, and longer intensive care and hospital stay [101]. Realtime monitoring with NIRS has also been utilised as a tool to detect cerebral malperfusion and change intraoperative strategies during aortic surgery [102]. However, data obtained in the form of rCSO_2_ needs to be interpreted with caution. Depth of penetration is limited to about 2 cm with only around a 60% contribution from the capillary bed, thus it only monitors anterior cerebral blood flow and is therefore limited in detecting embolic events and hypoperfusion in the basilar region of the brain [103, 104]. Its main use is therefore in detecting global and differential hemispheric changes. The data available from NIRS is subject to a number of intrinsic and extrinsic factors. Variables which are known to effect rCSO_2_ include haemoglobin concentration, arterial blood pressure, temperature, systemic oxygen saturations, and pCO_2_ [105] all of which may be altered in ATAAD surgery. Hypotension, anaemia, reduced oxygen saturation, and reduced pCO_2_ as a result of hyperventilation may all result in reduced rCSO_2_. These variables need to be corrected to accurately interpret the results obtained. Furthermore, there is a lack of predefined values for warning or intervention associated with changes in rCSO_2_ obtained with NIRS. It has been reported that levels of rCSO_2_ reductions of greater than 20% from baseline or rCSO_2_ levels less than 40–50% are associated with hypoxic ischemic neuronal injury [106, 107]. Clinically, it may be more relevant to monitor trends in rCSO_2_ rather than look at absolute values [105, 108]. In aortic arch surgery using SACP, rCSO_2 _ monitoring can facilitate decision making regarding the number of supra-aortic vessels requiring cannulation. In one report, the use of right axillary perfusion with innominate and left carotid clamping alone was associated with a prompt deterioration of left-sided rCSO_2_ signal in the majority of patients. This was correctable by the insertion of an additional perfusion cannula within the left carotid, following which left rCSO_2 _signals normalized [109]. As interpretation of rCSO_2_ signalling is usually based upon relative changes from a stable baseline, its main utility appears to be ensuring that an anticipated effect of perfusion and cooling is achieved without major hemispheric discrepancies. At present an absolute or relative value at which safe brain arrest can be commenced is not available. However, a unilateral progressive discrepancy in rCSO_2_ signalling, during the conduct of an ATAAD should raise major concerns that malperfusion is occurring. 

### 11.5. Jugular Venous Oxygen Saturation

Jugular bulb venous oxygen saturation (JbSVO_2_) monitoring may be used as an index of cerebral metabolic suppression. The normal JbSVO2 of a fully anaesthetised patient approximates 55–65% [110]. Provided oxygen delivery is normal with arterial SaO_2_ approximating 98–100%, the JbSVO_2_ reflects transcranial oxygen extraction. A low level is indicative of increased extraction or low flow and is a warning signal of inadequate perfusion. Reduction in jugular venous saturations (JbSjVO_2_) implies an imbalance between cerebral blood flow (CBF) and cerebral oxygen consumption (CMRO_2_). To achieve JbSjVO_2_ desaturation either CBF must be reduced or CMRO_2_ increased. JbSjVO_2_ does not correlate well with mixed venous oxygen saturations [111]. In aortic dissection, low initial JbSVO_2 _ may be an index of either low cardiac output due to tamponade and cardiac insufficiency or cerebral malperfusion and if uncorrected may be associated with worse outcome. 

During ATAAD surgery, cooling on bypass is a usual part of the brain protection strategy; during cooling, the JbSVO_2 _ rises. This is for two reasons, first as a direct consequence of hypothermic metabolic suppression and second due to increased haemoglobin avidity for oxygen in hypothermic conditions shifting the oxygen-haemoglobin dissociation curve leftwards and thereby reducing the P50. During CPB cooling, the JbSVO_2 _ has a linear inverse relationship with the cerebral metabolic rate for oxygen (CMRO_2_) relative to baseline [71]. Thus, as CMRO_2_ decreases, JbSVO_2 _ rises. A JbSVO_2_ ≥ 95% correlates with a CMRO_2_ of <20% of CMR at 37°C and once this level of JbSVO_2 _ is attained, the medical team can be assured that near-maximal clinically practicable metabolic suppression has been achieved and that it is safe to commit to a period of hypothermic circulatory arrest (subject to the time-limitations of HCA noted above). If a jugular bulb line is in place, the 95% JbSVO_2 _ threshold as a trigger to permit onset of HCA is a practical use of cerebral monitoring if isolated HCA without perfusion adjuncts is to be used as the mainstay of brain protection. However, there is little data in the literature to guide JbSVO_2_ monitoring targets if arrest is to be undertaken at a higher temperature with early institution of SACP. Interestingly, the precise relationship between JbSVO_2_ and NIRS rSCO_2_ has not been defined, although the two have been demonstrated to be closely linked in a physiological clinical study [112]. Cutoffs for commitment to HCA using rCSO_2_ are not available. Importantly, unilateral placement of a jugular bulb line will provide no information of differential hemispheric perfusion. It cannot therefore be used as a detection monitor for contralateral malperfusion. Its main role is in circumstances when bilateral carotid perfusion is assured and here it provides evidence of global perfusion. We are not aware of any studies using bilateral clinical bulb line monitoring.

The placement of a jugular bulb line is a relatively facile technique. The line is a simple central venous catheter and is inserted retrogradely through the internal jugular vein and positioned in the jugular bulb. As the line is advanced, gentle suction is applied on a connected syringe and once the jugular bulb is reached, a loss of resistance is felt within the syringe mechanism with increased ease of aspiration at low suction pressure. Usually, the left internal jugular vein is used for jugular bulb line placement assuming the right internal jugular will be used for the CVP and pulmonary artery catheter. Measurements of JbSVO_2_ can then be obtained either continuously or intermittently [113]. Although JbSVO_2_ allows monitoring of global oxygenation it does not provide regional information and is unlikely to detect focal events. Consequently, it is also not used to assess adequacy of SACP. If JbSVO_2_ and EEG are not available but HCA is the intended brain protection strategy, our protocol is to cool on 2.4 L·min^−1^ m^−2^ flow cardiopulmonary bypass, using a 7°C maximum temperature exchange difference for at least 50 minutes to a nasopharyngeal temperature of 15°C [79]. This duration and depth of cooling is known to be sufficient to achieve electrocerebral silence and attain JbSVO_2_ ≥ 95% in the vast majority of patients and represents a practical guide when additional monitoring is not available. 

After HCA, jugular venous saturations initially fall considerably and proportionately with the oxygen debt generated by the period of HCA. After a period of 5–15 minutes of rewarming, JbSVO_2_ levels then gradually rise as the cerebral metabolic debt is repaid until prebypass values are restored. Beyond this timepoint, jbSVO_2_ levels may fluctuate with changes in oxygen delivery and cardiac instability, reflecting the vulnerability of the recovering postischaemic brain to additional ischaemic insults. Although JbSjVO_2_ is a useful technique, setup time is significant and it may therefore not be a practical monitoring technique in the acute setting of ATAAD except in the most stable patients. 

## 12. Measures of Microcirculation and Regional Blood Flow

The majority of clinical monitoring tools used in the operating theatre and intensive care unit for assessment of cardiovascular performance evaluate global haemodynamics but these may not reflect derangements in regional organ perfusion. Tissue metabolic stress is dependent on microcirculatory flow and there are now a number of methods for examining microcirculatory perfusion that have been evaluated in the intensive care setting; these include microdialysis, laser doppler flowmetry, and side-stream dark imaging of mucosal capillary flow [114–116].

In terms of cardiac surgery, the most investigated and potentially most useful tool in monitoring of visceral blood flow is gastric tissue oxygenation and tonometry. Hypothermic CPB with nonpulsatile flow leads to somewhat predictable decreases in visceral blood flow measured by laser Doppler flowmetry and mucosal pH tonometry that could be of utility in the assessment and monitoring of visceral malperfusion during ATAAD surgery [117–121]. However, the use of such devices has to date been on a research basis and whether clinical implementation will prove worthwhile is not clear. 

There has been recent interest in the use of near infra-red spectroscopy as a noninvasive measure of distal limb perfusion. The NIRS signal has been validated as an accurate index of perfusion in clinical experiments of brachial artery occlusion and reperfusion and strain gauge plethysmography [122]. It has been used to detect lower limb arterial graft occlusion during vascular surgery [123] and during femoral artery cannulation for minimally invasive cardiac surgery, NIRS demonstrates reductions in tissue oxygen saturations on clamping which are normalised with distal leg perfusion and comparable to the noncannulated side [124]. The detection of acute lower limb ischaemia following femoral cannulation after aortic cross-clamping has also been reported utilising NIRS during aortic surgery [125], and this is a potential regional monitoring technique that could be of utility during ATAAD surgery. 

A further technique known as visible light spectroscopy has been used to detect gut mucosal oxygen saturation via probes placed either in the oesophagus or colon [126, 127]. This could potentially be a means of detecting regional mesenteric ischaemia in aortic surgery, although its use appears to be limited to case reports at the present time. 

## 13. Conclusion

The propensity for pre- and intraoperative malperfusion during ATAAD surgery necessitates a unique approach to cardiovascular anaesthetic monitoring. The organ at greatest risk is the brain, and monitoring strategies are designed to try and ensure that brain perfusion remains adequate at all times during surgery. However, at the present time, there is no monitoring device available that can measure brain perfusion in all regions intraoperatively and each part of the strategy has limitations. 

Our recommendations are evidence based where possible and where not are based upon considerable clinical experience. Individual circumstances including local facilities and expertise will dictate the particular monitoring strategy in each case and in reality evidence of clinical malperfusion is best demonstrated by a combination of techniques. 

Our current practice is to use bilateral radial arterial lines and on occasion an additional femoral line, intraoperative transoesophageal echocardiography and bilateral frontal lobe NIRS assessment of rCSO_2_ as standard in the emergency patient. In more stable patients this may be supplemented by other surrogate measures of cerebral metabolism such as transcranial Doppler and jugular bulb venous oximetry which we use, mainly, as research adjuncts. In addition we monitor nasopharyngeal temperature as a validated surrogate of direct brain temperature during cooling and rewarming [69] and adopt specific management protocols during these phases of the procedure [79]. Finally, all patients are monitored using pulmonary artery flotation catheters once separation form cardiopulmonary bypass has been achieved, thus enabling cardiac output to be optimised. We believe that the strategy we adopt provides as much information as currently possible in the ATAAD setting to detect and correct intraoperative malperfusion by adjustment of cannulation, anaesthetic, and perfusion techniques in an attempt to achieve improved outcomes in this difficult area. 

## Figures and Tables

**Table 1:** Essential and desirable monitoring techniques for use in repair of acute type A aortic dissection.

*Essential monitoring*
Electrocardiogram
Arterial oxygen saturations
Peripheral and core temperatures
Central venous pressure
Pre- and postarch arterial lines
Transoesophageal echocardiogram

*Desirable monitoring*

Pulmonary artery flotation catheter
Continuous intra-arterial blood gas monitor
Near infrared spectroscopy (cerebral and peripheral)
Jugular venous oxygen saturations
Transcranial Doppler
Electroencephalography

**Table 2:** Calculations of the safe duration of hypothermic circulatory arrest (HCA) based on 100% metabolic activity at 37°C and a safe HCA period of five minutes. Reproduced with permission from McCullough et al. [33].

Temperature (°C)	Cerebral metabolic activity (percentage of baseline)	Estimated safe duration of HCA (minutes (95% CI))
37	100	5
30	56 (52–60)	9 (8–10)
25	37 (33–42)	14 (12–15)
20	24 (21–29)	21 (17–24)
15	16 (13–20)	31 (25–38)
10	11 (8–14)	45 (36–62)
